# Self-confidence and perceived fatigue as predictors of performance satisfaction in trained swimmers

**DOI:** 10.3389/fpsyg.2026.1755167

**Published:** 2026-04-24

**Authors:** Alejandro López-Hernández, Juan Ángel Simón-Piqueras, José María González Ravé

**Affiliations:** 1 Sports Training Laboratory, Faculty of Sports Sciences of Toledo, University of Castilla-La Mancha, Toledo, Spain; 2 Faculty of Education of Albacete, University of Castilla-La Mancha, Albacete, Spain

**Keywords:** anxiety, perceived fatigue, performance prediction, psychological readiness, satisfaction, self-confidence, swimming

## Abstract

**Introduction:**

Pre-competitive anxiety and self-confidence are key psychological factors influencing athletic performance. While anxiety was traditionally seen as harmful, recent biopsychosocial models suggest its effects depend on athletes’ interpretation of arousal and their confidence level. This study examined whether pre-competitive self-confidence and perceived fatigue predicted post-competition satisfaction and results in trained youth swimmers, considering differences between individual and team formats.

**Methods:**

The sample consisted of 147 trained swimmers of national level (70 males and 77 females; Age = 16.1 ± 1.3 years) from Castilla-La Mancha, Spain. Participants took part in two major official competitions during the 2023–2024 season. Before and after each competition, they completed measures of somatic and cognitive anxiety, self-confidence, perceived fatigue, and satisfaction with their performance, using the CSAI-2R and complementary questionnaires. Pearson correlations and multiple regression analyses were conducted. Exploratory group comparisons were also performed across event distance (sprint, middle, long) and competition type (individual vs. team).

**Results:**

Self-confidence significantly predicted lower pre-competition perceived fatigue, and cognitive anxiety showed a small additional negative effect. In turn, both self-confidence and perceived fatigue predicted post-competition satisfaction, whereas anxiety dimensions did not contribute unique variance. An exploratory model showed that perceived fatigue was the dominant perceptual predictor of objective performance (Δ%), with no independent effects of confidence or anxiety. No differences in psychological variables or performance emerged across distance groups or competition formats.

**Conclusion:**

Pre-competition self-confidence and perceived fatigue are key psychological indicators of swimmers’ perceived performance and satisfaction, while anxiety plays only a minor role. Monitoring these perceptual states may help anticipate athletes’ readiness and guide targeted psychological interventions during competition.

## 1 Introduction

Anxiety is a normal and adaptive emotional reaction to situations that people perceive as threatening or challenging. It prepares the body and mind to face possible danger and is therefore essential for survival. However, when the anxious response becomes too intense or occurs too often, it loses its functional character and may interfere with well-being, decision-making, or performance in demanding contexts [American Psychiatric Association [APA], 2022].

In sports, *competitive anxiety* refers to an unpleasant emotional state triggered by competitive situations. It usually involves both somatic (physiological) and cognitive (worry-related) components that can influence athletic performance (Hussain et al., 2021). Over the last decade, theories have moved toward a more biopsychosocial view, describing anxiety as the product of interactions between biological, cognitive, and contextual elements (Makovac et al., 2023).

Within this approach, two main forms of anxiety are usually identified: *state anxiety*, a short-term emotional response to a particular stressful event, and *trait anxiety*, a relatively stable tendency to experience anxiety in various situations (Chakrabarty and Thakur, 2024). Alongside these, self-confidence stands out as another key psychological factor in sport. It refers to an athlete’s belief in their ability to deal effectively with competition demands, acting as a kind of cognitive shield that reduces perceived threat and helps maintain control. Beyond its emotional effects, self-confidence also influences behavior during competition—for example, how athletes make decisions, focus attention, and cope with difficulties. Generally, more confident athletes use more task-focused strategies and manage stress better (Amaro and Brandão, 2023). In this sense, self-confidence mediates how athletes interpret stressors, use their psychological resources, and transform emotions into effective or maladaptive actions.

The connection between anxiety, self-confidence, and performance has long been recognized in sport psychology. According to Bandura’s self-efficacy theory (1997), belief in one’s capability to perform a task regulates both emotional and behavioral responses. Thus, self-confidence often works as a protective factor against anxiety, helping reduce perceived threat and favoring adaptive coping (Paersch et al., 2025; Besharat and Pourbohlool, 2011).

Classic meta-analyses by Woodman and Hardy (2003) and Craft et al. (2003) provided solid empirical evidence for this relationship. Woodman and Hardy reviewed more than forty studies and concluded that confidence was a stronger predictor of performance than cognitive anxiety. They pointed out that confident athletes tend to interpret symptoms of anxiety—such as tension or arousal—as signs of readiness rather than danger. Similarly, Craft et al. (2003), analyzing data from the CSAI-2, found that the effect of anxiety on performance depends largely on how the athlete interprets their activation and on situational factors such as sport type or competition level.

More recent research has added that variables like personality, gender, and age also play a role in how anxiety is experienced. Female athletes usually report higher somatic and cognitive anxiety and somewhat lower self-confidence than males (Amaro and Brandão, 2023; Kemarat et al., 2022). Younger athletes also tend to score higher in anxiety and lower in confidence compared with older or more experienced peers (Martínez-Gallego et al., 2022). Moreover, traits such as neuroticism or extraversion predict anxiety intensity, while emotional stability and conscientiousness are linked to greater self-confidence and better coping skills (Gabrys and Wontorczyk, 2023; Zubić, 2021).

A recent study with Spanish swimmers emphasized that individual psychological resources—including confidence, resilience, and emotional regulation—are critical to maintaining consistent performance during competitions (López-Hernández et al., 2025). Overall, these findings show that anxiety and confidence depend not only on situational demands but also on stable personal characteristics that shape how athletes perceive and handle pressure.

These ideas fit well with the *direction and intensity* theory of anxiety proposed by Jones and Swain (1992) 1995. This model suggests that anxiety is not necessarily harmful—its effects depend on whether the athlete sees it as helpful or as a threat. Empirical studies support this: for instance, Vesković and Stamenković (2022) found that both the intensity and the *direction* (facilitative or debilitative) of anxiety were important predictors of basketball performance (Ntoumanis and Biddle, 2000). Likewise, Bosshard and Gomez (2024) showed that interpreting activation as readiness can improve concentration and reaction time, whereas perceiving it as a threat can disrupt attention and harm performance.

There is also growing evidence that self-confidence and emotional control involve neurobiological processes. They influence cortical activity, functional connectivity, and attention networks during performance (van Noordt and Segalowitz, 2012; Humińska-Lisowska, 2024). This broader view has led to more comprehensive models where anxiety is no longer seen only as a barrier but as a flexible component that can even be useful when accompanied by confidence and good emotional regulation (Woodman and Hardy, 2003).

At the same time, it is worth remembering that psychological responses do not occur in isolation. Factors like training periodization, tapering, or the physical preparation phase may influence the anxiety–performance relationship (Lochbaum et al., 2022). These contextual elements can either reduce pressure effects or enhance optimal activation.

Two studies have recently contributed to this line of work. Dalamitros et al. (2023) tested the effects of a post-activation potentiation (PAP) protocol on psychophysiological variables in swimmers. Although no clear changes in pre-competitive anxiety or performance were found, the authors noted that anxiety responses might not be easily detected outside real competition, and that highly trained athletes could need stronger stimuli to activate optimally. Similarly, Fortes et al. (2017) observed that higher cognitive and somatic anxiety correlated with lower post-competition heart rate variability, suggesting an autonomic imbalance that, while not directly impairing results, could hinder recovery between races.

Based on this evidence, the present study aimed to examine the predictive relationships among pre-competitive psychological states in trained youth and junior swimmers. Specifically, we investigated how pre-competitive levels of self-confidence, anxiety, and perceived fatigue were associated with post-competition satisfaction and objective race outcomes. Replicating these relationships in real competition contexts is essential, as most previous research has been conducted in training environments or with mixed-sport samples. Furthermore, integrating perceived pre-competition fatigue—a variable rarely included in predictive models—provides an opportunity to extend and validate the findings of Craft et al. (2003), Lochbaum et al. (2022), Fortes et al. (2017), and Hakim et al. (2022) within a sport-specific and ecologically valid setting. In this context, it was hypothesized that (H1) different pre-competition levels of self-confidence, anxiety, and perceived fatigue would significantly predict athletes’ post-competition satisfaction with performance, and (H2) that self-confidence and anxiety would significantly predict athletes’ perceived fatigue before competition.

Although the primary aim of this study was to evaluate the predictive capacity of these psychological states, we also conducted exploratory comparisons to examine whether anxiety, self-confidence, fatigue, or satisfaction differed across competition formats (individual vs. team). This secondary objective was included to ensure alignment with earlier literature suggesting that contextual variables may modulate pre-competitive emotional responses. Together, these analyses contribute real-world evidence on how psychological factors interact in competitive swimming, offering insight into when pre-competition states may support or hinder athletes’ perceived performance and subsequent satisfaction.

## 2 Materials and methods

### 2.1 Participants

The study sample included 147 swimmers competing at the national level, all belonging to clubs from the autonomous community of Castilla-La Mancha (Spain). Of these, 70 were men and 77 women, aged between 14 and 18 years (*M* = 16.1; SD = 1.3). Every participant had at least 3 years of competitive experience at the national level and was active during the 2023–2024 season. Following the classification proposed by McKay et al. (2022), these swimmers can be categorized as Tier 3 athletes (Highly Trained/National Level), which aligns with previous regional studies conducted in similar high-performance contexts. To provide additional detail on competitive level, average Aqua Points were as follows: male swimmers aged 14–15 years averaged 478 points, female swimmers of the same age group averaged 461 points, male swimmers aged 16–18 years averaged 591 points, and female swimmers in that category averaged 588 points. These scores are consistent with national-level performance standards in youth swimming, confirming the sample’s representativeness within high-performance developmental categories.

With respect to event distance, around half of the male swimmers specialized in sprint races ( ≤ 100 m), 41% in middle-distance (200–400 m), and about 9% in long-distance events ( ≥ 800 m). Among females, the distribution was slightly different: 39% focused on sprinting, 46% on middle-distance, and 15% on long-distance disciplines. This overall pattern reflects the aerobic–anaerobic specialization typically seen in national-level swimming in Spain.

Participation in the study was voluntary. All swimmers—and their legal guardians in the case of minors—signed an informed consent form before data collection began. The research followed the ethical principles of the Declaration of Helsinki and received approval from the institutional Ethics Committee (Ref. CEIS-739611-H7M1). A summary of sample characteristics is shown in Table 1.

**TABLE 1:** Model 1 (pre-competitive fatigue).

Summary								
Model	*R*	*R* ^2^	Adjusted *R*^2^	RMSE	AIC	BIC	DW statistic	DW*p*
M0 (Intercept only)	0.000	0.000	0.000	0.934	400.130	406.111	1.601	.015
M1 (Full model)	0.483	0.233	0.217	0.827	367.154	382.106	1.776	.164
ANOVA								
**Source**	**Sum of squares**	**df**	**Mean Square**	***F***	***p***			
Regression	29.67	3	9.891	14.47	< 0.001			
Residual	97.73	143	0.683					
Total	127.40	146						
Coefficients								
**Predictor**	**B**	**SE**	**β (std)**	***t***	***p***	**Tolerance**	**VIF**	
Intercept	4.261	0.409	–	10.42	< 0.001	–	–	
Cognitive anxiety	–0.197	0.095	–0.168	–2.075	0.040	0.821	1.218	
Self-confidence	–0.614	0.096	–0.472	–6.397	< 0.001	0.984	1.016	
Somatic Anxiety	0.119	0.133	0.072	0.893	0.373	0.827	1.209	

Own elaboration. M1 includes cognitive anxiety, self-confidence, and somatic anxiety.

### 2.2 Procedure

The study followed a within-subject, descriptive, and correlational design in which each swimmer completed psychological assessments in real competitive conditions. All participants were evaluated prior to official competition events to examine the relationship between pre-competitive psychological states and subsequent performance satisfaction. In addition, athletes completed a post-competition psychological measure assessing satisfaction with their performance, which served as the primary post-event outcome variable. Although predictive analyses were the main focus of the study, exploratory comparisons across competition types (individual vs. team) were also conducted to ensure that potential contextual differences did not influence the observed psychological patterns.

To assess anxiety and self-confidence, the Competitive State Anxiety Inventory-2 Revised (CSAI-2R) was used, originally developed by Cox et al. (2003) was adapted to Spanish sport people by Andrade Fernández et al. (2007). This instrument is one of the most widely used tools in sport psychology to measure pre-competitive state anxiety and athlete self-confidence. The CSAI-2R consists of 17 items divided into three subscales:

- Cognitive Anxiety (5 items), which evaluates worries and negative performance-related thoughts (e.g., *“I am concerned about losing”).*
- Somatic Anxiety (7 items), which assesses physiological and activation symptoms such as tension or nervousness (e.g., *“My body feels tense”).*
- Self-Confidence (5 items), which measures perceived control and belief in one’s ability to perform successfully (e.g., *“I am confident I can meet the challenge”).*

Responses were collected using a 4-point Likert scale (1 = *not at all* to 4 = *very much so*). The Spanish version of the Competitive State Anxiety Inventory-2 Revised (CSAI-2R) employed here has shown strong psychometric properties with athlete samples, reporting internal consistency values above.80 across its three subscales—cognitive anxiety, somatic anxiety, and self-confidence (Andrade Fernández et al., 2007; Fernández, 2008). Previous confirmatory factor analyses also supported the original three-factor model, showing adequate fit indices (χ^2^/df < 2.0, CFI ≥ .93, TLI ≥ .91, RMSEA ≤ .06), which supports its factorial validity for young athletes. In this study, Cronbach’s α values were 0.84 for cognitive anxiety, 0.82 for somatic anxiety, and 0.88 for self-confidence, suggesting good reliability. These results are consistent with prior findings in youth swimmer populations (López-Hernández et al., 2025).

Perceived fatigue was measured using a simple, single-item question commonly used in competition settings to quickly assess fatigue before performance (e.g., “How fatigued do you feel right now?”), rated from 1 (no fatigue) to 10 (maximum fatigue). This subjective indicator is widely applied in applied sport psychology as an easy and reliable way to capture physical and mental readiness under real competition conditions. Participants completed the survey digitally by scanning a QR code that linked to a Google Forms questionnaire, which they accessed on their own mobile devices to ensure anonymity and confidentiality of responses. All instruments were administered in real competition settings approximately ten minutes before each athlete’s race, in a quiet area near the pool. This timing allowed for a consistent and ecologically valid measurement of pre-competitive emotional states—including cognitive anxiety, somatic anxiety, self-confidence, and perceived fatigue—while minimizing external distractions or interference.

In addition to the CSAI-2R, swimmers again rated their perceived fatigue after competition using the Rating of Perceived Exertion/Fatigue (RPE-F) scale (Ferreira et al., 2014). Although the post-event fatigue rating was not used as a predictor, it allowed us to describe perceptual changes associated with the competitive effort and relate them to psychological states. Performance data (personal best, season best, race time) were collected to compute performance variation percentages (Δ%), which served as an objective performance indicator in the exploratory analysis. All procedures were conducted under controlled and standardized conditions, supervised by the research team and coaches.

### 2.3 Statistical analysis

To calculate the minimum required sample size, a priori power analysis was conducted using the G*Power 3.1 software. Considering an expected medium effect size (*f* = 0.15), a significance level of *p* = 0.05, a desired statistical power of (1–β) = 0.80, and three predictors, the minimum required sample size was 77 participants. The final sample included 147 swimmers, exceeding this threshold.

All statistical analyses were performed using JASP software (version 0.19.3 for Apple Silicon, University of Amsterdam, The Netherlands). Normality of the data was verified through the Shapiro–Wilk test, confirming that all variables met normal distribution assumptions [W(147) > 0.978, *p* > 0.05].

Bivariate relationships among variables were examined using Pearson’s correlation coefficients. Subsequently, the extent to which swimmers’ pre-competition anxiety and self-confidence predicted pre-competition perceived fatigue, and the predictive capacity of self-confidence, perceived fatigue, and anxiety on post-competition satisfaction. A third exploratory regression model was used to examine whether pre-competition perceptual variables predicted objective performance (Δ%). For all regression models, assumptions of linearity, non-collinearity, independence, homoscedasticity, and approximate normality of residuals were verified.

In addition, exploratory one-way ANOVAs were performed to assess whether anxiety, self-confidence, perceived fatigue, satisfaction, or Δ% performance differed across event distance groups (sprint, middle, long) or competition formats (individual vs. team). These analyses were included to ensure that contextual variability did not account for the patterns observed in the predictive models.

## 3 Results

### 3.1 Pearson’s correlation coefficient

**FIGURE 1:**
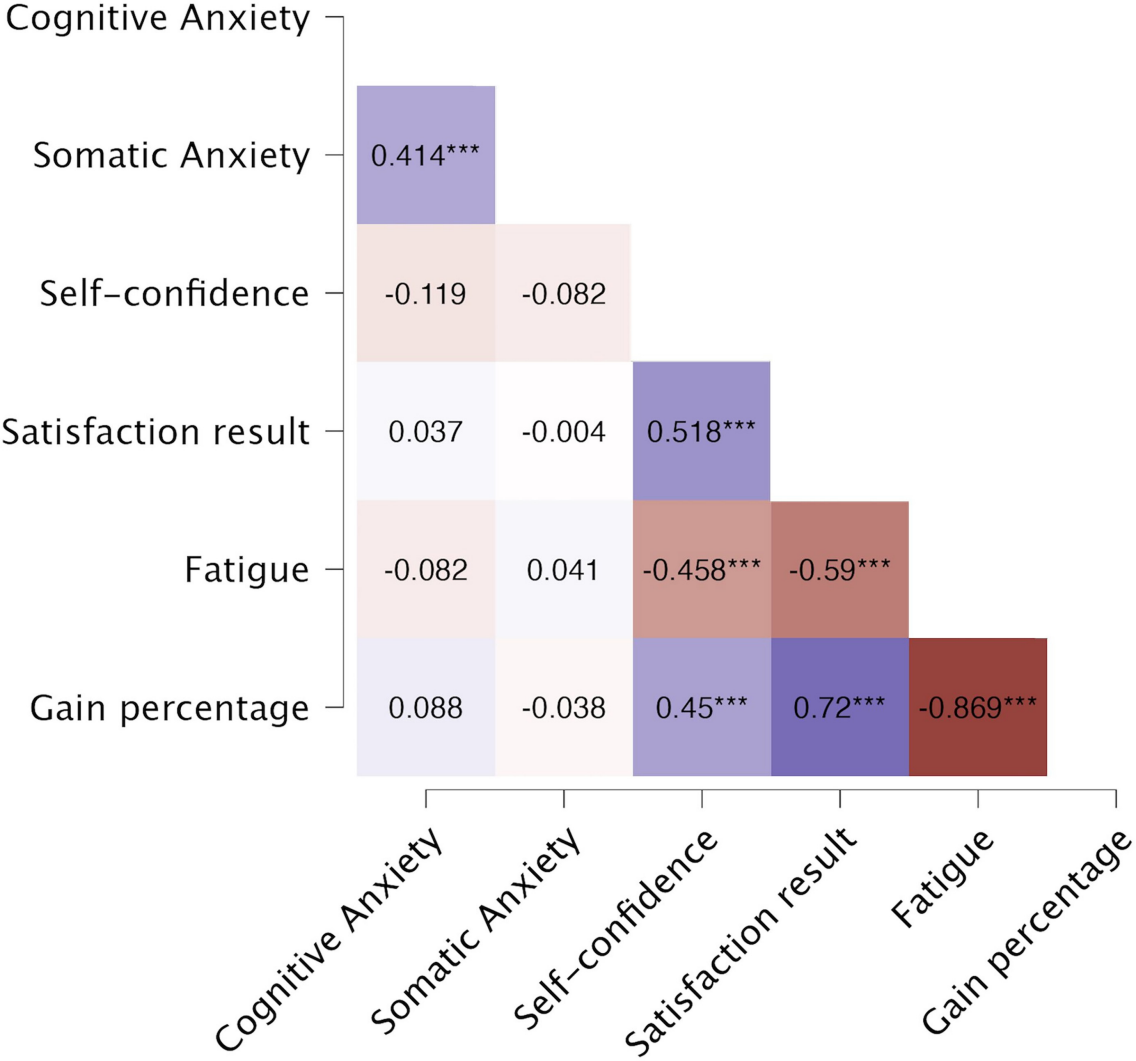
Pearson’s correlation coefficient heatmap. Own elaboration. ***P < (0.001).

The correlation matrix showed significant positive associations between somatic and cognitive anxiety, and between self-confidence and post-competition satisfaction. Significant negative correlations were found between self-confidence and perceived fatigue, and between perceived fatigue, satisfaction, and Δ% performance. No substantial correlations were observed between anxiety dimensions and the outcome variables included in the regression models (Figure 1).

However, no significant correlations were observed between either form of anxiety (cognitive or somatic) and the key variables used in the predictive models (perceived fatigue, post-competition satisfaction and gain percentage).

### 3.2 Linear regression analysis to establish whether self-confidence, cognitive anxiety and somati anxiety predicts the perception of precompetitive fatigue

**FIGURE 2:**
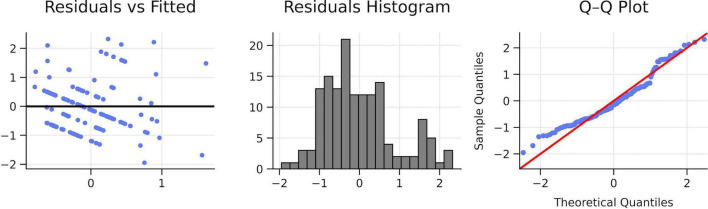
Diagnostic plots. Own elaboration. Residuals show no major deviations (homoscedasticity correct; approximate normality), supporting the adequacy of the linear model.

First, the rest of the previous requisitives were evaluated for the application of the multiple linear regression analysis (Table 2). The requirements of non-collinearity (VIF < 10, Tolerance > 0.1), Independence (Durbin—Watson 1.5–2.5), homoscedasticity verified through the prediction vs. residual scatter plot and finally through the Q-Q graphs the normality of errors (Figure 2) were met. The model was significant, *R*^2^ = 0.233, adjusted *R*^2^ = 0.217, *F*(3, 143) = 14.47, *p* < 0.001. Self-confidence significantly predicted lower fatigue [β = –0.472, SE = 0.074, 95%CI (–0.618, –0.326), *p* < 0.001]. Cognitive anxiety showed a small additional negative effect (β = –0.168, SE = 0.081, 95%CI (–0.328, –0.008), *p* = 0.040]. Overall, lower self-confidence and higher cognitive anxiety were associated with greater pre-competitive fatigue.

**TABLE 2:** Model 2 (Satisfaction).

Summary								
Model	*R*	*R* ^2^	Adjusted *R*^2^	RMSE	AIC	BIC	DW statistic	DW *p*
M0 (Intercept only)	0.000	0.000	0.000	0.996	430.442	436.423	1.843	.210
M1 (Full model)	0.653	0.427	0.411	0.764	371.065	389.018	1.861	.251
ANOVA								
**Source**	**Sum of squares**	**df**	**Mean square**	***F***	***p***			
Regression	62.25	4	15.563	26.49	< 0.001			
Residual	83.55	142	0.589					
Total	145.80	146						
Coefficients								
**Predictor**	**B**	**SE**	**β (std)**	***t***	***p***	**Tolerance**	**VIF**	
Intercept	5.847	0.377	–	15.51	< 0.001			
Self-confidence	0.524	0.119	+0.321	4.40	< 0.001	0.767	1.304	
Fatigue	–0.689	0.119	–0.441	-5.78	< 0.001	0.828	1.207	
Cognitive anxiety	–0.033	0.081	–0.039	–0.41	0.684	0.753	1.328	
Somatic anxiety	0.037	0.083	+0.026	0.45	0.654	0.750	1.334	

Own elaboration. M1 includes self-confidence, fatigue, cognitive anxiety, and somatic anxiety.

**FIGURE 3:**
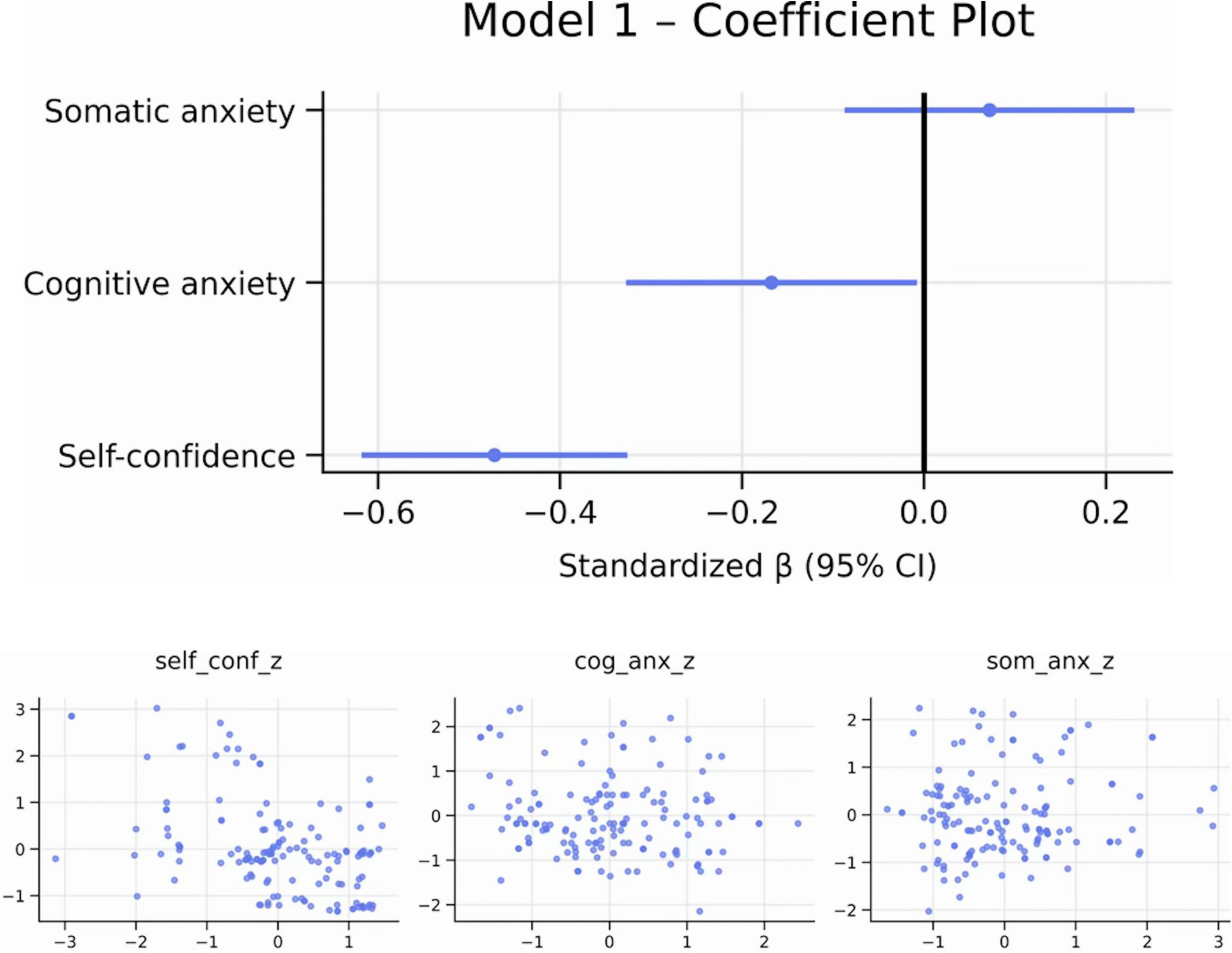
Coefficient and added-variable plots. Own elaboration. Coefficient plot: Lower self-confidence and higher cognitive anxiety uniquely predict greater pre-competitive fatigue. Somatic anxiety shows no meaningful effect. Added-Variable Plots: Self-confidence: clear negative unique association. Cognitive anxiety: small negative unique effect. Somatic anxiety: residual pattern near zero (no predictive value).

**FIGURE 4:**
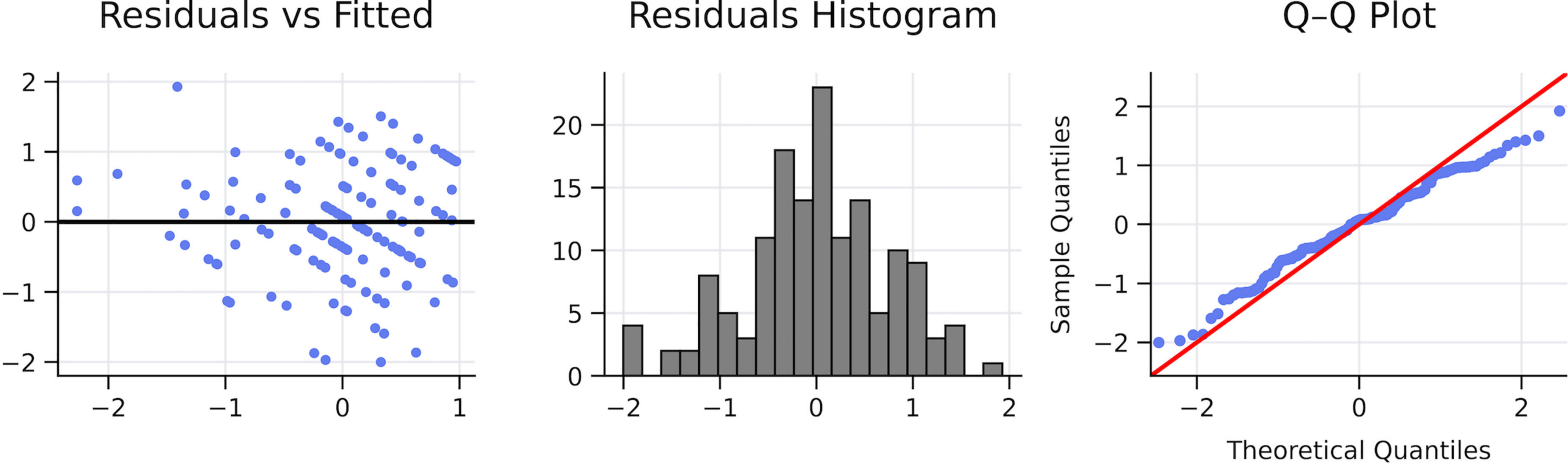
Diagnostic plots. Own elaboration. Residual diagnostics did not indicate major violations of assumptions: there were no serious problems with heteroscedasticity, residuals approximated normality, and no concerning autocorrelation patterns were observed.

The regression model explained 23.3% of the variance in pre-competitive fatigue, indicating a moderate overall predictive capacity. Among the predictors, self-confidence emerged as the dominant factor, accounting for approximately 22% of unique variance in fatigue. In contrast, cognitive anxiety contributed only a small but statistically significant proportion of unique variance (about 3%). Somatic anxiety did not show capacity predictive (Figures 3, 4 and Table 1).

### 3.3 Linear regression analysis to establish whether cognitive anxiety, somatic anxiety, self-confidence and precompetitive fatige predict the satisfaction result

A multiple linear regression was conducted to examine whether self-confidence, perceived fatigue, cognitive anxiety, and somatic anxiety, predicted post-competition satisfaction. The overall model was significant, *R*^2^ = 0.427, adjusted *R*^2^ = 0.411, *F*(4,142) = 26.49, *p* < 0.001, indicating a substantial predictive contribution of pre-competitive states to athletes’ satisfaction with their performance. Assumption checks showed no meaningful autocorrelation (Durbin–Watson = 1.86) and no issues of multicollinearity (all VIF ≤ 1.31).

Self-confidence emerged as a significant positive predictor of satisfaction [β = +0.321, SE = 0.073, 95% CI (+0.178, +.465), *p* < 0.001], indicating that athletes who approached the event with higher confidence tended to report greater post-competition satisfaction. In contrast, pre-competitive fatigue was a strong negative predictor [β = –0.441, SE = 0.073, 95% CI (–0.585, –0.298), *p* < 0.001], showing that athletes who felt more fatigued before competing reported lower satisfaction with their performance. Neither cognitive anxiety nor somatic anxiety showed significant direct effects (*p*s > 0.68), suggesting that anxiety levels did not uniquely explain additional variance in satisfaction when confidence and fatigue were considered. Overall, these results indicate that pre-competitive self-confidence and perceived fatigue are the principal psychological predictors of athletes’ satisfaction with their performance, while anxiety dimensions do not contribute direct explanatory value in this context.

**FIGURE 5:**
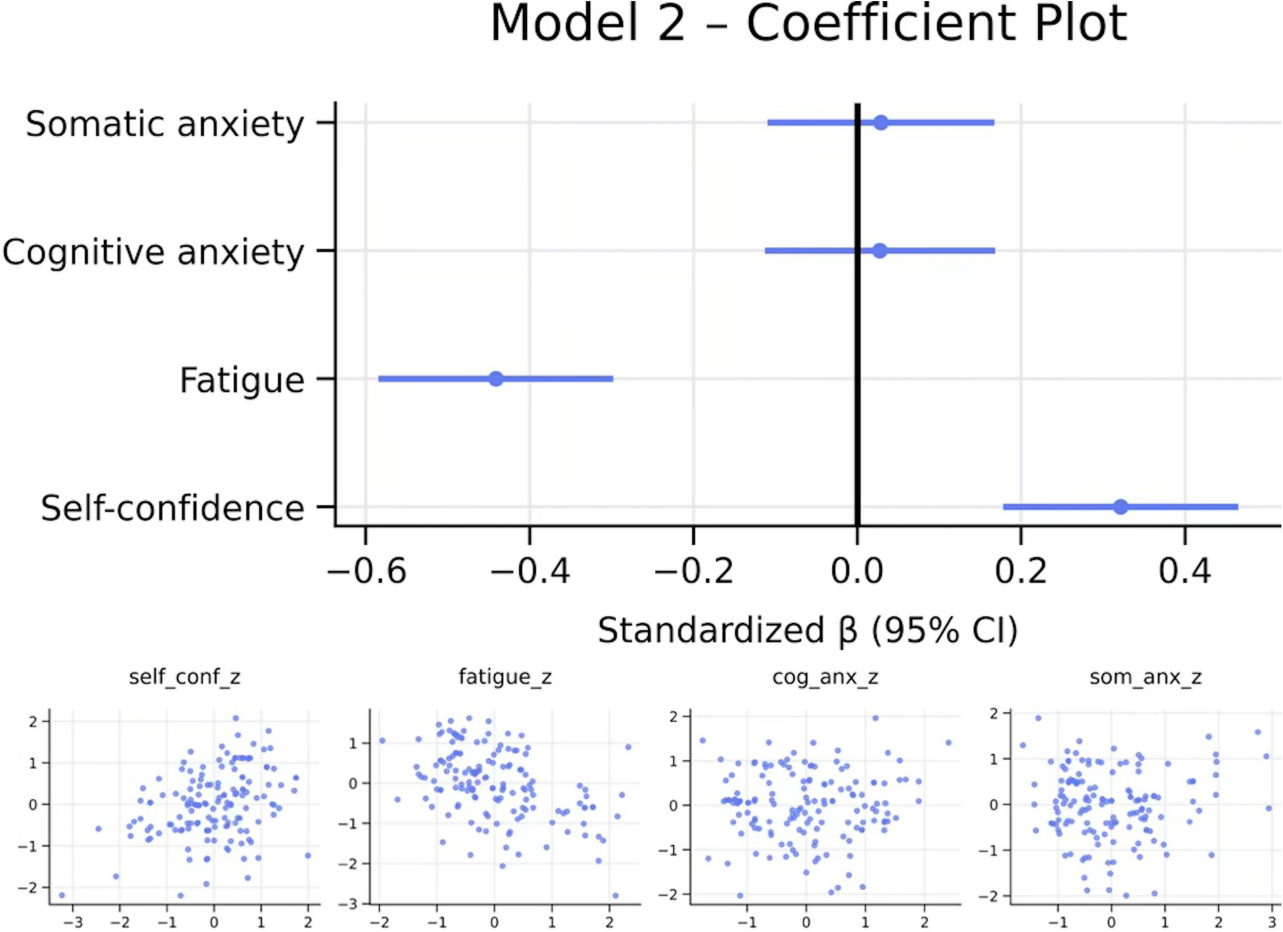
Coefficient and added-variable plots. Own elaboration. Self-confidence predicted higher post-competition satisfaction, whereas pre-competition perceived fatigue predicted lower satisfaction. Cognitive and somatic anxiety did not show significant unique effects once confidence and fatigue were considered.

The model explained 42.7% of the variance in post-competition satisfaction, indicating a substantial predictive contribution of pre-competitive psychological states. Fatigue was the strongest unique predictor (≈20% unique variance), followed by self-confidence (≈10% unique variance). In contrast, cognitive and somatic anxiety each accounted for less than 1% of unique variance and did not reach significance. Overall, pre-competitive fatigue and confidence are the key determinants of satisfaction, whereas anxiety dimensions add no meaningful direct predictive value (Figure 5 and Table 2).

### 3.4 Additional analysis: exploratory model of psychological predictors of Δ% performance

To complement the primary regression models, we conducted an exploratory analysis examining whether pre-competition psychological variables predicted objective performance (Δ%). This model was not part of the original hypotheses and is therefore presented for descriptive purposes only. The full regression results are shown in Table 3. The aim was to assess the extent to which athletes’ pre-competitive perceptual states (self-confidence, fatigue, cognitive anxiety, and somatic anxiety) were associated with subsequent race performance, providing supplementary insight into the prospective links between psychological readiness and objective outcomes.

**TABLE 3:** Model 3 (Δ%).

**Summary**								
Model	*R*	*R* ^2^	Adjusted *R*^2^	RMSE	AIC	BIC	DW statistic	DW *p*
M1 (Full model)	0.872	0.760	0.753	0.805	360.299	378.241	1.812	0.239
ANOVA								
**Source**	**Sum of squares**	**df**	**Mean square**	***F***	***p***			
Regression	290,9	4	72.74	l112.3	< 0.001			
Residual	92.01	142	0.648					
Total	383.0	146						
Coefficients								
**Predictor**	**B**	**SE**	**β (std)**	***t***	***p***	**Tolerance**	**VIF**	
Intercept	0.000	0.041		0.00	1.000			
Self-confidence (z)	0.072	0.047	+0.072	1.53	.128	0.73	1.37	
Fatigue (z)	–0.833	0.047	–0.833	–17.73	< 0.001	0.72	1.39	
Cognitive anxiety (z)	0.034	0.046	+0.034	0.74	0.459	0.84	1.19	
Somatic anxiety (z)	–0.013	0.045	–0.013	–0.28	0.782	0.86	1.16	

Own elaboration.

**FIGURE 6:**
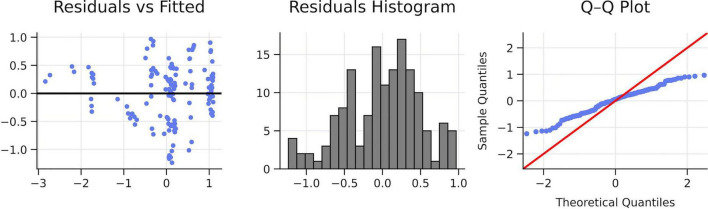
Diagnostic plots. Own elaboration. Residual diagnostics indicated no substantial heteroscedasticity. Residuals were approximately normally distributed. Autocorrelation diagnostics did not reveal any problematic patterns.

**FIGURE 7:**
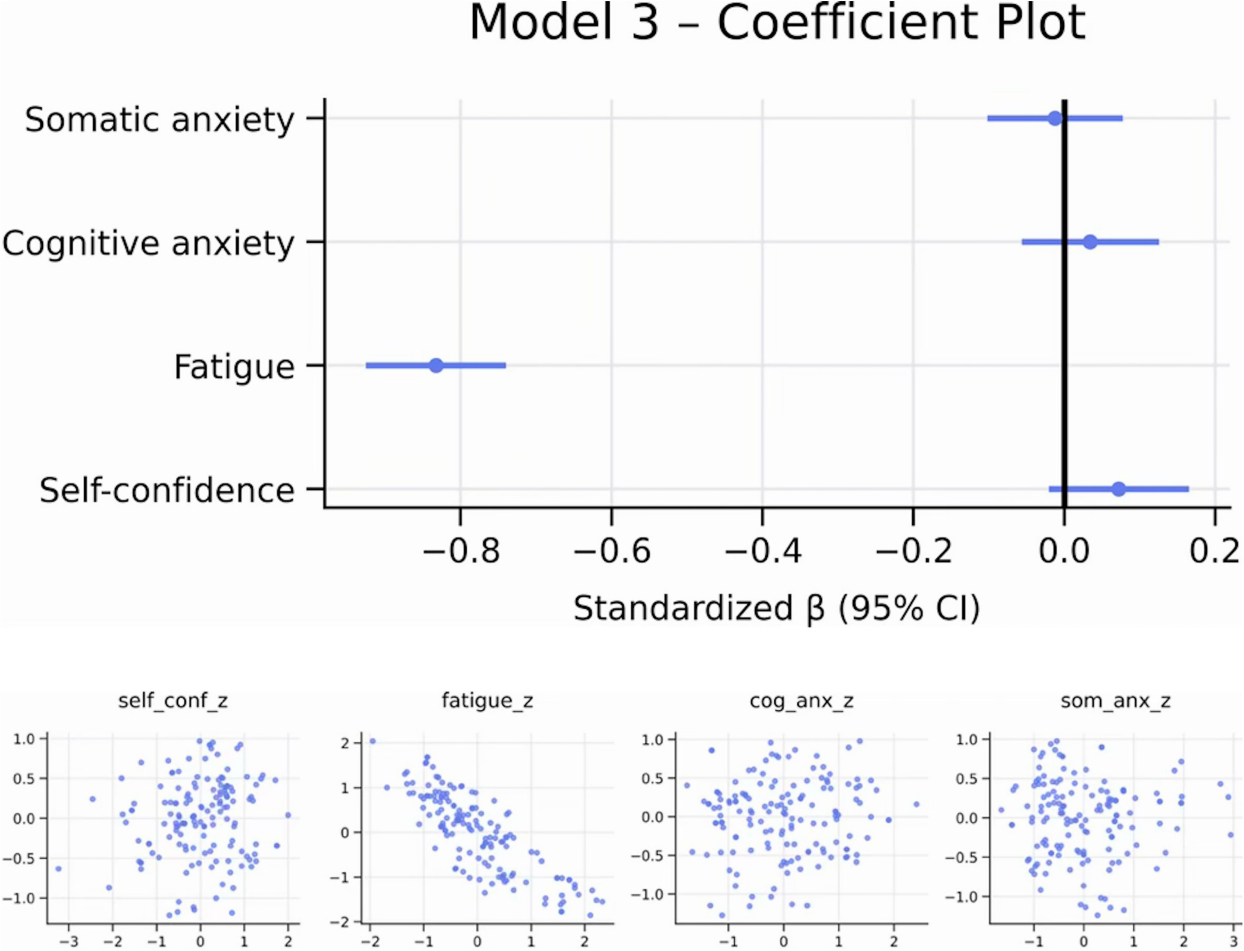
Coefficient and added-variable plots. Own elaboration. Perceived pre-competitive fatigue is the absolute predictor of performance (β ≈ –0.83). Self-confidence and anxieties do not have an independent effect.

The regression model predicting objective performance (Δ%) from pre-competition perceptual variables was highly significant (*R*^2^ = 0.760). Pre-competitive fatigue was the strongest predictor (β = –0.83, *p* < 0.001), indicating that athletes who felt more fatigued before competing obtained markedly poorer objective results. Self-confidence showed a small positive, but non-significant (β≈ +0.72) in the alternative model), whereas anxiety dimensions did not display significant direct effects. These findings suggest that pre-competitive fatigue are key perceptual indicator of subsequent objective performance (Figures 6, 7).

### 3.5 One-way ANOVA comparisons across event distance and competition format

To verify that the psychological predictors identified in the regression models operated consistently across different competitive settings, we conducted exploratory one-way ANOVAs comparing groups by event distance (sprint, middle, long) and competition type (individual vs. team). This allowed us to determine whether contextual factors might influence pre-competitive states or performance outcomes.

Across both distance groups (Sprint, Middle, Long) and competition type (individual vs. team), no significant differences were found for any psychological variable or Δ% performance. This suggests that pre-competitive states and performance outcomes remained consistent across event formats and distances. The detailed ANOVA results are presented in Table 4.

**TABLE 4:** One-way ANOVAs by distance group and competition type.

Variable	F (distance)	p (distance)	F (competition type)	p (competition type)
Cognitive anxiety	1.29	0.277	0.33	0.566
Somatic anxiety	0.79	0.454	0.38	0.538
Self-confidence	1.79	0.171	0.98	0.324
Fatigue (perceived)	1.86	0.159	0.29	0.588
Satisfaction	1.36	0.261	1.74	0.189
Δ% performance	1.75	0.178	0.54	0.463

Own elaboration.

## 4 Discussion

This study examined the relationship between pre-competitive anxiety, self-confidence, perceived fatigue, and satisfaction with performance in youth and junior swimmers across two competition formats (individual and team). The findings provide a nuanced understanding of how psychological variables interact to shape performance outcomes in real competitive contexts.

These results reinforce that optimal athletic performance does not depend on the mere absence of anxiety but on maintaining a dynamic balance among arousal, self-confidence, and emotional regulation. This interaction aligns with biopsychosocial and IZOF frameworks, which posit that athletes perform best when anxiety is interpreted as a facilitative signal rather than a threat (Barlow et al., 2023; Jones and Swain, 1995; Woodman and Hardy, 2003). Psychological skills training should therefore focus not on eliminating anxiety, but on reframing it as a functional component of performance through strategies such as self-regulation, cognitive restructuring, and confidence enhancement. Recent meta-analytic evidence supports this view, indicating a moderate positive association between self-confidence and sport performance [*r* = 0.25, 95% CI (0.19–0.30)], with stronger effects in individual sports, where self-reliance and emotional control are crucial (Lochbaum et al., 2022). Overall, this suggests that athletes with greater emotional stability and confidence are more capable of remaining within their Individual Zone of Optimal Functioning (IZOF), transforming physiological arousal into concentration and adaptive coping responses that enhance performance (Ruiz et al., 2017).

Importantly, the results revealed that both research hypotheses were partially supported. With regard to H1, self-confidence and perceived fatigue significantly predicted performance satisfaction, whereas anxiety did not contribute to the predictive model. For H2, self-confidence significantly predicted perceived fatigue, but again anxiety did not show a predictive effect. These findings suggest that, in this sample of trained youth swimmers, self-confidence exerts a more robust role than anxiety in shaping fatigue perception and satisfaction outcomes. Beyond its statistical significance, performance satisfaction represents a crucial psychological indicator of perceived success and well-being in competitive sport.

In the present study, satisfaction with performance reflects not only the athlete’s evaluation of results but also their subjective sense of control, effort, and fulfillment after competition. This outcome provides valuable insight into how swimmers cognitively and emotionally appraise their own performance, independent of objective times or rankings. Athletes with higher self-confidence and lower perceived fatigue tended to report greater satisfaction, suggesting that psychological readiness may shape post-event evaluations as much as physical outcomes (Jekauc et al., 2023). Therefore, performance satisfaction can be understood as both a reflection of immediate emotional response and a marker of long-term motivation and engagement in sport.

This study demonstrates that athletes’ self-confidence significantly predicts their pre-competitive perceived fatigue, showing a moderate and inverse relationship between both variables: higher self-confidence is associated with lower perceived fatigue prior to competition (Jekauc et al., 2023, Moen et al., 2019). Moreover, both self-confidence and perceived fatigue jointly predict athletes’ post-competition satisfaction with performance, with self-confidence emerging as a direct and positive predictor, and perceived fatigue as an inverse predictor. These results indicate that knowing athletes’ pre-competition levels of self-confidence and perceived fatigue allows for a reliable prediction of perceived performance quality and post-event satisfaction.

In addition to these findings, the exploratory regression model predicting Δ% performance showed that pre-competition perceived fatigue was the only meaningful psychological predictor of objective race outcomes. Neither self-confidence nor the anxiety dimensions contributed unique variance once fatigue was considered, indicating that swimmers’ subjective sense of readiness aligns closely with their actual performance.

This predictive capacity provides valuable applied implications. It enables pre-emptive psychological interventions before competition when negative forecasts are detected—specifically, when self-confidence is low or perceived fatigue is high. Such an approach reinforces the connection between psychological variables and fatigue perceptions, enhancing understanding of how mental states influence readiness and competitive responses, even when fatigue manifests subjectively.

The findings support the theoretical framework proposed by Woodman and Hardy (2003), suggesting that anxiety and self-confidence do not influence performance linearly, but rather through their interaction and cognitive interpretation. Within this framework, self-confidence emerges as a key modulatory factor, capable of transforming anxious activation into functional energy and buffering fatigue perception—thereby contributing to greater psychological stability and performance satisfaction.

Furthermore, exploratory ANOVAs showed no differences in psychological states or performance across competition formats or event distances. This indicates that the predictive patterns identified in the regression models are robust across different competitive contexts.

From an applied standpoint, these results highlight the importance of developing pre-competition monitoring protocols that systematically assess self-confidence and perceived fatigue as indicators of mental readiness. Such evaluations could allow coaches and sport psychologists to anticipate psychological deviations and apply targeted emotional or motivational adjustments prior to competition.

First, the results confirmed that self-confidence acts as a key protective factor against perceived fatigue and post-competition dissatisfaction. Multiple regression models showed that self-confidence significantly predicted perceived fatigue (*R*^2^ ≈ 0.21) and, together with perceived fatigue, explained a substantial proportion of variance in satisfaction with performance (*R*^2^ ≈ 0.42). This finding aligns with self-efficacy theory (Bandura, 1997) and the meta-analyses by Woodman and Hardy (2003) and Craft et al. (2003), which identify self-confidence as a stronger predictor of athletic performance than cognitive anxiety. Confidence not only reduces perceived threat but also facilitates positive reinterpretations of physiological activation and enhances the use of effective coping strategies. Recent research further supports these relationships, showing that higher self-confidence and self-management behaviors are positively associated with team satisfaction and athletic performance (Kang and Lim, 2017), while greater confidence also predicts higher life satisfaction and lower psychological fatigue among athletes (Grebner et al., 2024; Rinaldy et al., 2022).

In contrast, neither cognitive nor somatic anxiety predicted satisfaction, and only cognitive anxiety showed a small, negative association with pre-competition perceived fatigue, suggesting that these variables might play more of a moderating or mediating role rather than a strictly causal one. This finding is consistent with the direction and intensity theory of anxiety (Jones and Swain, 1992, 1995), which argues that anxiety itself is not necessarily harmful—what really matters is how athletes interpret it, whether as facilitative or debilitative. Recent work supports this view, showing that athletes with stronger self-esteem and more adaptive coping skills can sometimes use anxiety as a driving, even motivating, force instead of an obstacle (Moroianu and Popescu, 2023). Similar patterns have been observed in younger athletes, where emotional control and experience seem to help transform anxiety into focus and readiness rather than tension (Beenen et al., 2025). In other words, swimmers with more competitive experience may have learned to reinterpret arousal as a sign that they are prepared to perform, not a signal of threat. These results are also in line with the framework proposed by Woodman and Hardy (2003), which suggests that anxiety affects performance through its interaction with self-confidence and the athlete’s interpretation of arousal. Moderate anxiety levels can, in fact, sharpen attention, increase physiological readiness, and boost competitive motivation—particularly in individual sports, where athletes tend to bear pressure more personally. At the same time, it’s important to remember that other non-psychological factors, such as training periodization, tapering, or specific preparation cycles, might have also influenced performance during the competitions analyzed (Lochbaum et al., 2022). These contextual variables can shape how anxiety impacts performance, sometimes buffering its negative side while enhancing the energizing effects of optimal activation.

In swimming, where both individual and team pressure fluctuate depending on the event, understanding pre-competitive anxiety is especially relevant. Tools such as the Competitive State Anxiety Inventory-2 (CSAI-2) have been widely used to measure cognitive and somatic anxiety along with self-confidence, making it possible to examine these psychological processes under authentic competitive conditions (Craft et al., 2003; Silva et al., 2019).

Recent findings further confirm that psychological readiness and physiological states are closely linked in swimming. For example, heart rate variability has been connected with pre-competition anxiety and readiness to perform (Ortigosa-Márquez et al., 2017). Likewise, mental fatigue has been shown to negatively affect both perceived effort and actual performance in time-trial events among competitive swimmers (Penna et al., 2017), with similar results reported at the national level (de Lima-Junior et al., 2025). Furthermore, anxiety intensity often fluctuates with training load and can predict variations in performance, as higher workloads tend to increase state anxiety while slightly lowering self-confidence (Aouani et al., 2024).

Moreover, recent research emphasizes that both psychological resources and coaching style can buffer the negative effects of competitive anxiety. Supportive coaching environments that promote confidence and emotional regulation have been linked to higher performance and lower anxiety in adolescent swimmers (Sridana et al., 2024). Similarly, psychological strengths such as resilience and self-efficacy predict better performance through reduced stress and improved self-regulation (Wang et al., 2025).

From a practical standpoint, these findings highlight the importance of combining psychological and physiological monitoring to improve performance outcomes. Coaches and sport psychologists might benefit from focusing on the systematic strengthening of athletes’ self-confidence, helping them reinterpret anxiety-related activation in a positive way, and managing fatigue perceptions more effectively. Mental preparation programs could include emotional regulation strategies, psychological routines adjusted to tapering periods, and continuous tracking of fatigue and satisfaction levels. Likewise, exposing swimmers in a controlled way to competitive pressure during training could teach them to channel anxiety as a useful source of energy rather than as a limiting factor—ultimately improving focus, readiness, and overall satisfaction during real competition.

Even so, several limitations should be kept in mind when considering these results. This study was conducted with a regional sample of youth and junior swimmers from central Spain, so the findings may not fully apply to athletes from other countries or competition levels. Replicating the research with larger and more diverse groups—covering different cultural, geographic, and performance backgrounds—would help strengthen its external validity. It would also be useful to include additional variables such as the direction of anxiety (facilitative vs. debilitative), coping styles, or physiological indicators like heart rate variability, lactate concentration, or markers of neuromuscular fatigue, to gain a deeper understanding of the mechanisms involved in pre-competitive emotional states.

Future studies could also use more advanced analytical approaches, such as mediation or moderation models, to clarify the specific role that self-confidence plays within the anxiety–fatigue–performance relationship. These models could help determine whether perceived fatigue acts as a mediator between readiness and satisfaction, adding nuance to existing theories of sport performance. Examining how different competitive formats—for instance, high-pressure individual events versus team competitions—affect these dynamics would also help explain how athletes regulate emotions and maintain performance under varying conditions.

Finally, from an applied angle, these results may serve as a practical guide for coaches and sport psychologists, especially in contexts where physiological monitoring is not possible. In such cases, simple self-report indicators like perceived fatigue and self-confidence can serve as accessible, low-cost tools for anticipating athletes’ readiness and satisfaction. By identifying potential risk profiles before competition, practitioners can intervene early through cognitive and emotional regulation strategies, helping reduce fatigue perceptions, boost confidence, and encourage more adaptive competitive mindsets. In this sense, even basic perceptual measures can be extremely useful for managing day-to-day performance, particularly in youth sport settings where technological resources are often limited.

Expanding research in these directions will deepen understanding of how athletes regulate emotions and fatigue under competitive stress, supporting the design of evidence-based psychological preparation programs that foster optimal arousal, confidence, and emotional control for sustained high performance.

## 5 Conclusion

This study demonstrates that athletes’ self-confidence significantly predicts their pre-competitive perceived fatigue, showing a moderate and inverse relationship between both variables. In addition, cognitive anxiety showed a small negative association with perceived fatigue, although its contribution was notably weaker than that of self-confidence. Moreover, both self-confidence and perceived fatigue jointly predicted athletes’ post-competition satisfaction, with self-confidence emerging as a positive predictor and perceived fatigue as a negative one. Finally, the exploratory analysis revealed that perceived pre-competition fatigue was also the strongest perceptual predictor of objective performance (Δ%). Taken together, these results indicate that monitoring athletes’ pre-competition levels of self-confidence and perceived fatigue provides valuable insight into both perceived performance quality and actual race outcomes, offering practical indicators of readiness in competitive swimming.

## Data Availability

The raw data supporting the conclusions of this article will be made available by the authors, without undue reservation.
